# ﻿A new species and updates on *Paravelia* Breddin, 1898 (Hemiptera, Heteroptera, Veliidae) in Brazil

**DOI:** 10.3897/zookeys.1238.142181

**Published:** 2025-05-15

**Authors:** Evaldo Alves Joaquim, Juliana Mourão dos Santos Rodrigues, Felipe Ferraz Figueiredo Moreira, Leandro Lourenço Dumas

**Affiliations:** 1 Laboratório de Entomologia, Instituto Oswaldo Cruz, Fundação Oswaldo Cruz, Avenida Brasil, 4365, 21040-360, Rio de Janeiro, RJ, Brazil Fundação Oswaldo Cruz Rio de Janeiro Brazil; 2 Laboratório de Insetos Aquáticos, Departamento de Biologia Animal, Instituto de Ciências Biológicas e da Saúde, Universidade Federal Rural do Rio de Janeiro, Rodovia BR-465, Km 7, 23890-000, Seropédica, RJ, Brazil Universidade Federal Rural do Rio de Janeiro Seropédica Brazil; 3 Programa de Pós-graduação em Biologia Animal, Universidade Federal Rural do Rio de Janeiro, Rodovia BR-465, Km 7, 23890-000, Seropédica, RJ, Brazil Universidade Federal do Rio de Janeiro Seropédica Brazil

**Keywords:** Aquatic insects, geographical distribution, Gerromorpha, Neotropical region, semiaquatic bugs, taxonomy, water striders

## Abstract

*Paravelia* Breddin, 1898 is the most speciose genus of the subfamily Veliinae (Hemiptera, Heteroptera, Gerromorpha, Veliidae). It has gone through revisions since the 1990s, with several species being transferred to other genera, and currently contains 46 species distributed from Mexico to Argentina, 35 of which occur in Brazil. Here, we describe *P.intervalensis***sp. nov.** based on material obtained during an expedition carried out in 2023 in the Parque Estadual Intervales, state of São Paulo. The new species can be distinguished from congeners by the the following: body length between approximately 5.1–5.5 mm; head, thorax, and abdomen (except posterior margin of sternum VII) not covered by small black denticles; humeral angle not spinose; forewings without bubble-like structures, with a pair of elongated yellow maculae basally and a small, diamond-shaped, white macula apically; venter of abdomen not covered by punctations; male abdominal sternum VII without projections or lobes; male proctiger without conical process at base, without lateral projections approximately at middle of length; and male paramere with a dorsal notch. We also present new distribution records of *P.luederwaldti* Rodrigues & Moreira, 2016 and provide an updated key to the *Paravelia* recorded from Brazil. This study increases the number of species of *Paravelia* known in Brazil to 36.

## ﻿Introduction

*Paravelia* Breddin, 1898 is the most speciose genus of the subfamily Veliinae (Hemiptera, Heteroptera, Gerromorpha, Veliidae). It contains 46 species distributed from Mexico to Argentina, 35 of which occur in Brazil (Moreira 2024; Rodrigues and Moreira 2024). These semiaquatic bugs inhabit a wide range of lentic and lotic freshwater habitats, including emergent vegetation, surfaces of large rocks, logs, and roots in the water, as well as humid terrestrial environments (Mazzucconi et al. 2009). Alongside *Microvelia* Westwood, 1834, they are also the only gerromorphans inhabiting phytotelmata (Polhemus and Polhemus 1991).

*Paravelia* has gone through revisions since the 199’s, with several species being transferred to other genera (Polhemus and Polhemus 1993; Polhemus et al. 2019; Polhemus 2021; Rodrigues and Moreira 2024). Currently, it is characterized by following combination of features: mesoacetabulum and metasternum lacking lateral tubercles; tarsomere II of the middle leg usually 4–5 times longer than tarsomere I; pretarsus of the middle and hind legs with setae-shaped arolia and two falcate claws; and the macropterous form usually exhibiting two maculae on each forewing, one basally and the other apically (Rodrigues and Moreira 2016a, 2022, 2024).

In this study, we describe a new species of *Paravelia* from the state of São Paulo, Brazil. We also present new distribution records of *P.luederwaldti* Rodrigues & Moreira, 2016 and provide an updated key to the *Paravelia* recorded from Brazil.

## ﻿Methods

### ﻿Study area

Parque Estadual Intervales (PEI) is a conservation unit (CU) in the state of São Paulo, southeastern Brazil, that was established in 1995. It forms an ecological corridor together with five other contiguous CUs, representing one of the largest remnants of Atlantic Forest (Furlan and Leite 2008; Ribeiro et al. 2009). Currently, PEI encompasses an area of 41,704 ha in the Serra do Paranapiacaba mountain range, spreading through the municipalities of Eldorado Paulista, Guapiara, Iporanga, Ribeirão Grande, and Sete Barras. Elevations range from 60 to 1,100 m, which, combined with the humid climate, generates a wide range of environments. Two major hydrographic basins originate from the highest areas within PEI: Ribeira de Iguape river basin, which flows towards the coast, and Paranapanema river basin, which flows inland (Furlan and Leite 2008).

### ﻿Sampling and taxonomy

**Figure 1.**
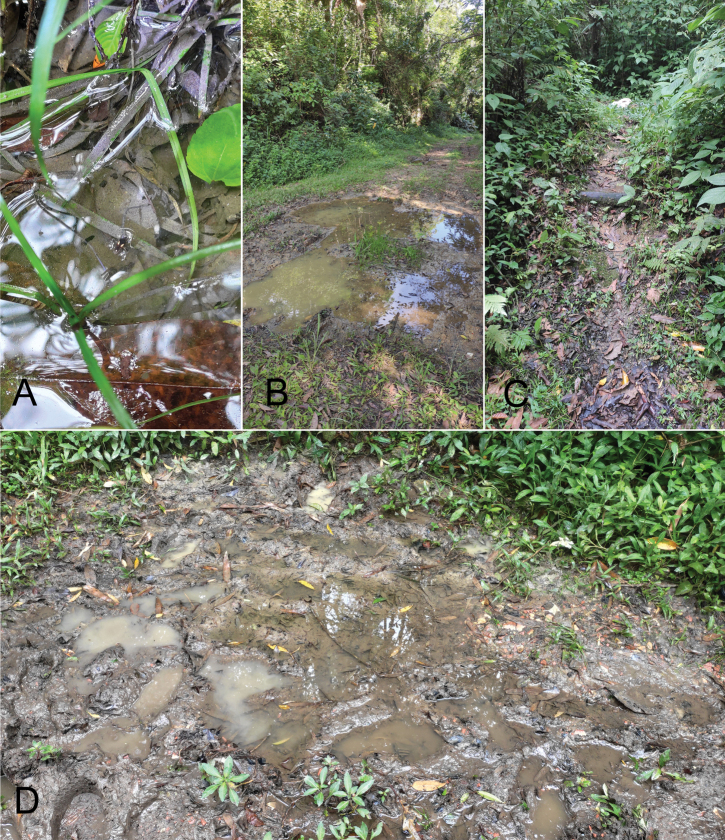
Photographs of the collecting localities in the Parque Estadual Intervales, São Paulo state, Brazil. **A, D** Puddles where *P.intervalensis* sp. nov. was collected **B, C** puddles where *P.luederwaldti* Rodrigues & Moreira, 2016 was collected.

We conducted two expeditions to PEI in November and December 2023 under permit from the Sistema Integrado de Gestão Ambiental of the state of São Paulo (#5695/2023). We obtained geographic coordinates of the collecting localities with a GPS receiver: (GPS Status and Toolbox, MobiWIA Ltd, Budapest, Hungary). We collected specimens using small sieves in temporary rain pools formed along trails (Fig. 1). Specimens are deposited in the Coleção Entomológica do Instituto Oswaldo Cruz, Rio de Janeiro, Brazil (**CEIOC**).

Terminology, description, and measurements follow the standards established in the latest revision of the genus (Rodrigues et al. 2014). We obtained photographs using a Leica M205 C stereomicroscope coupled with a digital camera and the Leica LAS imaging system. We produced maps using QGIS version 3.34.11. The distribution of each species is according to Moreira (2024) and abbreviations of Brazilian states are according to the official standard (IBGE 2024).

Abbreviations used for measurements are as follows: body length (**BL**), head length (**HL**), head width through eyes (**HW**), length of antennomeres I–IV [without intersegmental pieces] (**ANT I**, **ANT II**, **ANT III**, **ANT IV**), maximum eye width (**EYE**), pronotum length on midline (**PL**), pronotum width (**PW**), length of foreleg segments (**FORELEG**), length of midleg segments (**MIDLEG**), length of hindleg segments (**HINDLEG**), femoral length (**FEM**), tibial length (**TIB**), length of tarsomeres I–III (**TAR I**, **TAR II**, **TAR III**). All measurements are given in millimeters.

## ﻿Results and discussion

### ﻿New species

#### ﻿ Paravelia intervalensis sp. nov.

Taxon classificationAnimaliaHemipteraVeliidae

58C5CB9D-E9A4-57E6-B0E2-E289CDD09BCB

https://zoobank.org/F19F5762-5779-4E91-AC7B-37AE0E65AFFC

Figs 2 , 3 , 4 , 6

##### Type material.

***Holotype.*** Brazil – **São Paulo** • Iporanga, Parque Estadual Intervales, poça de chuva na trilha; 24°16'29.6"S, 48°24'54.4"W; 23.XI.2023; L.L. Dumas, J.M.S. Rodrigues & R.P.R. Canejo leg.; macropterous ♂, CEIOC 83571. ***Paratypes.*** Same data as holotype; 17 macropterous ♂, 12 macropterous ♀, CEIOC 83572.

##### Macropterous male.

**Figure 2.**
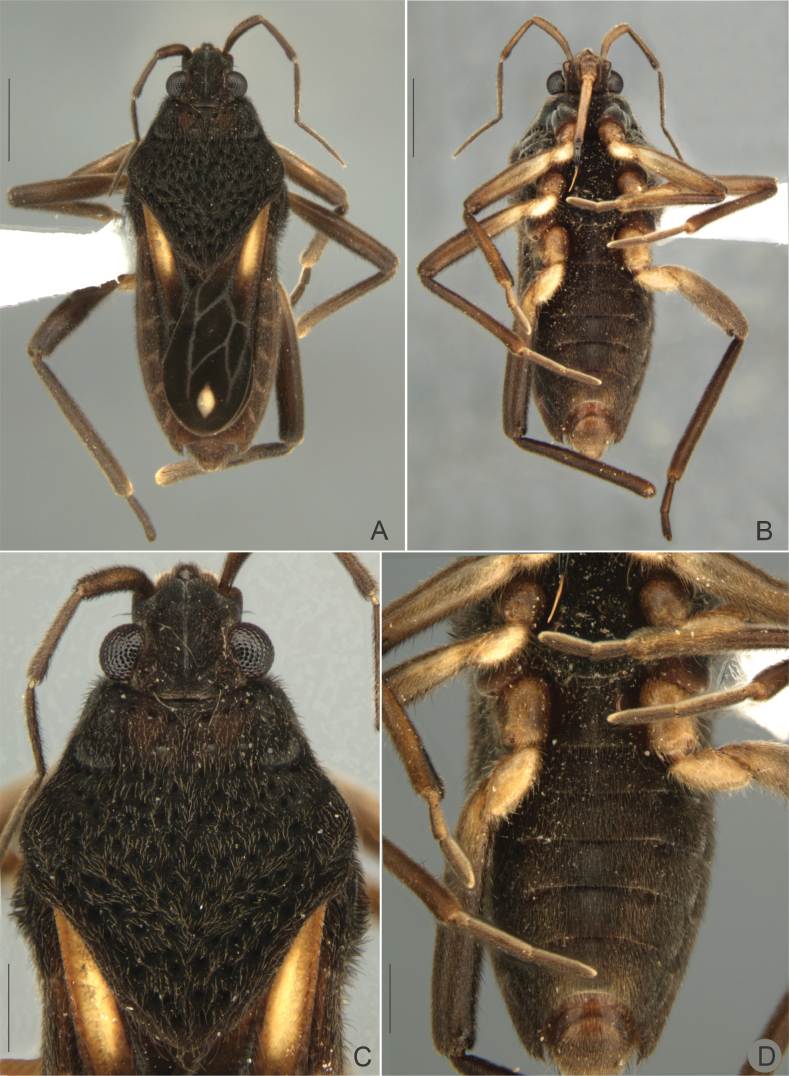
*Paraveliaintervalensis* sp. nov., holotype, macropterous male. **A** Dorsal view **B** ventral view **C** head, pronotum and part of the anterior wing, dorsal view **D** abdomen and part of the legs, ventral view. Scale bars: 0.5 mm (**C, D**); 1 mm (**A, B**).

**Figure 3.**
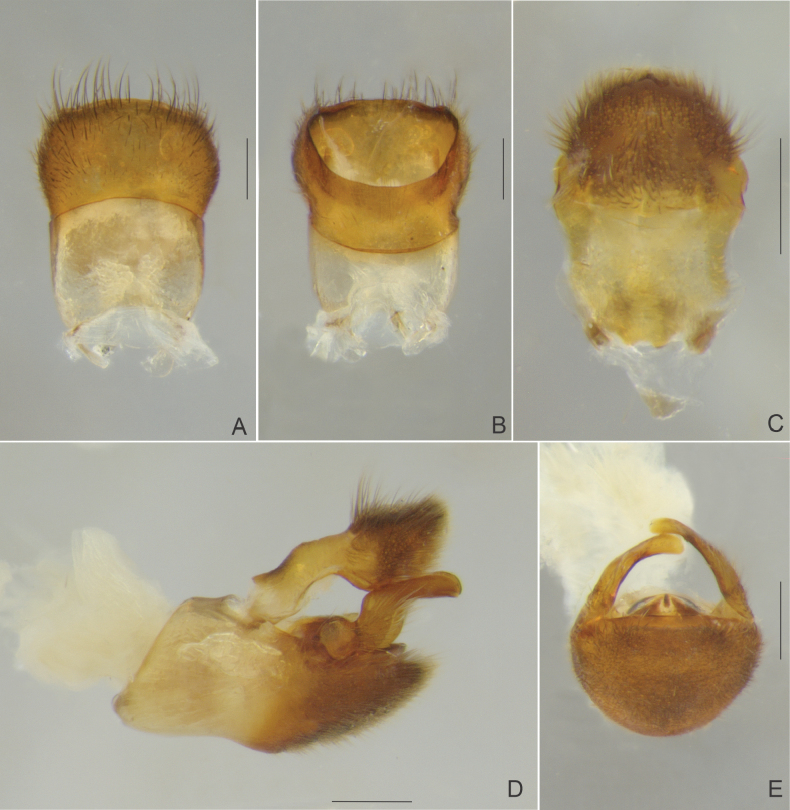
*Paraveliaintervalensis* sp. nov., male terminalia. **A, B** Abdominal segment VIII **A** dorsal view **B** ventral view **C** proctiger, dorsal view **D, E** genital capsule **D** lateral view **E** anterior view. Scale bars: 0.2 mm.

(Figs 2, 3) BL 5.12–5.18; HL 0.50–0.57; HW 0.95–1.00; ANT I 0.59–0.60; ANT II 0.59–0.60; ANT III 0.65–0.68; ANT IV 0.74–0.77; EYE 0.20–0.23; PL 1.80–1.85; PW 1.85–1.90; FORELEG, FEM 1.28–1.34; TIB 1.25–1.31; TAR I 0.07–0.10; TAR II 0.24–0.26; TAR III 0.38–0.41; MIDLEG, FEM 1.55–1.68; TIB 1.55–1.60; TAR I 0.08–0.12; TAR II 0.58; TAR III 0.52–0.56; HINDLEG, FEM 1.96–2.03; TIB 2.28–2.36; TAR I 0.08–0.15; TAR II 0.38–0.45; TAR III 0.45–0.55.

General color dark brown to black. Head black. Antenna brown to dark brown; antennomeres I–II brown, with base and apex dark brown. Labium with two basal articles brown; article III yellow laterally, brown medially; distal segment blackish. Pronotum black. Thoracic sterna black. Acetabula dark brown laterally; yellowish-brown mesally. Forewing black, with an elongated yellow macula reaching humeral angle and slightly surpassing posterior margin of pronotum; at apex, a small diamond-shaped white macula, smaller than basal macula (Fig. 2A); veins whitish. Coxae brown, with yellow marks. Trochanters yellow. Femora dark brown, with yellow mark at base of ventral surface; fore femur with yellowish mark at middle of dorsal surface. Tibiae brown, with yellow mark at apex. Tarsi dark brown. Abdominal laterotergites light brown mesally, dark brown laterally; intersegmental areas with yellowish marks. Venter of abdomen, dark brown; terminalia lighter.

Head covered by fine golden pubescence intermixed with elongate black and golden setae; dorsal midline impressed, shiny, posteriorly with pair of oblique, impressed, shiny lines and pair of indentations near mesal eye margins. Buccula and jugum without black denticles. Ocular setae present. Antenniferous tubercle developed, shiny. Antenna covered by golden pubescence and elongate golden setae scattered on segments II–IV; antennomere I robust, curved laterally; II thicker than III–IV.

Pronotum subpentagonal, covered by fine golden pubescence intermixed with elongate black setae; anterior lobe with row of rounded punctations adjacent to anterior margin; posterior lobe covered by rounded punctations, these larger towards apex; humeral angle slightly elevated; posterior angle slightly tapered, apex rounded (Fig. 2C, D). Forewings leaving apex of abdominal segment VII exposed, with four closed cells; veins on basal half with small golden setae. Proepimeron with rounded punctations. Meso- and metapleura with scattered rounded punctations. Prosternum with a row of four rounded punctations anteriorly. Meso- and metasterna with two pairs of small tubercles meeting centrally at intersegmental region. Legs densely covered by short, appressed, pale setae and elongate, brownish setae. Hind femur without spines.

Abdominal laterotergites covered by golden pubescence; elevated, without black denticles; last produced posteriorly. Abdominal sterna covered by fine golden pubescence and elongate dark brown setae; the latter concentrated on lateral margins; II compressed laterally and bearing weak longitudinal carina anteriorly; VII without projections or nodules (Fig. 2C); posterior margin evenly concave, with robust black denticles posterolaterally. Abdominal segment VIII with fine, golden pubescence on apical 2/3 intermixed with elongate, dark-brown setae dorsolaterally (Fig. 3A, B). Proctiger with elongate golden setae on apical half, without spines (Fig. 3C). Paramere (Fig. 3D, E), in lateral view, notched on dorsal surface, sinuous, with elongate golden setae and rounded apex (Fig. 3D).

##### Macropterous female.

**Figure 4.**
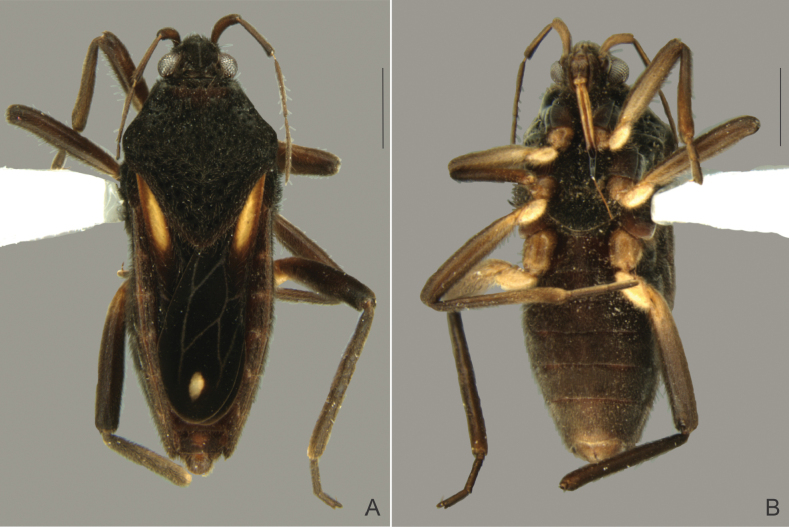
*Paraveliaintervalensis* sp. nov., paratype, macropterous female. **A** Dorsal view **B** ventral view. Scale bars: 1 mm.

(Fig. 4) BL 5.37–5.44; HL 0.60–0.66; HW 1.08–1.15; ANT I 0.54–0.60; ANT II 0.60–0.66; ANT III 0.72–0.80; ANT IV 0.80–0.86; EYE 0.20–0.28; PL 1.85–2.00; PW 1.87–1.94; FORELEG, FEM 1.25–1.28; TIB 1.25–1,29; TAR I 0.07–0.10; TAR II 0.20–0.25; TAR III 0.38–0.42; MIDLEG, FEM 1.60–1.68; TIB 1.76–1.80; TAR I 0.07–0.10; TAR II 0.48–0.56; TAR III 0.48–0.50; HINDLEG, FEM 1.80–1.85; TIB 2.26–2.30; TAR I 0.06–0.08; TAR II 0.50–0.54; TAR III 0.48–0.52.

General color and structure as in brachypterous male. Body longer; forewings leaving posterior margin of abdominal mediotergite VI exposed; abdomen more robust, with laterotergites more elevated.

##### Etymology.

The new species is named after Parque Estadual Intervales, the type locality.

##### Discussion.

*Paraveliaintervalensis* sp. nov. can be distinguished from the other species of the genus by the following combination of features: BL ~5.1–5.5; head, thorax, and abdomen (except posterior margin of sternum VII) not covered by small black denticles; humeral angle not spinose (Figs 2A, C, 4A); forewings without bubble-like structures, with a pair of elongated yellow maculae basally and a small diamond-shaped white macula apically (Figs 2A, C, 4A); yellow maculae reaching humeral angles and white macula far from apex of forewings (Figs 2A, C, 4A); venter of abdomen not covered by punctations (Figs 2B, D, 4B); male abdominal sternum VII without projections or lobes (Fig. 2D); male proctiger without conical process at base, without lateral projections approximately at middle of length (Fig. 3C); and male paramere with a dorsal notch in lateral view (Fig. 3D).

**Figure 5.**
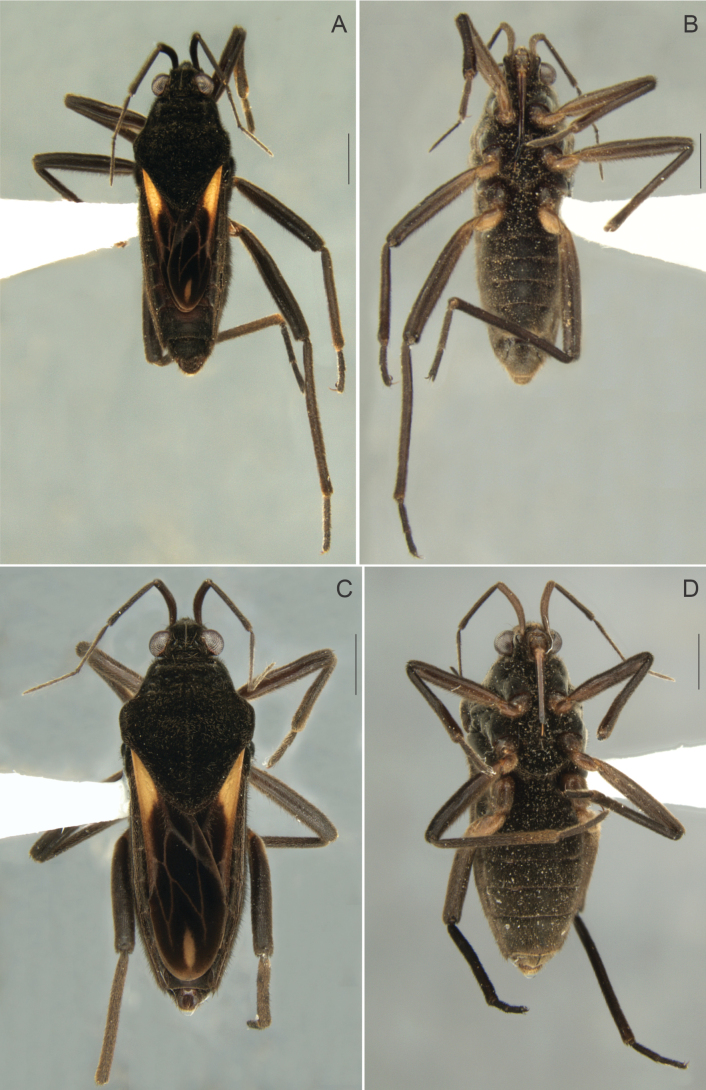
*Paravelialuederwaldti* Moreira & Rodrigues, 2016. **A, B** Brachypterous male **A** dorsal view **B** ventral view **C, D** submacropterous female **C** dorsal view **D** ventral view. Scale bars: 1 mm.

This species runs to couplet *P.luederwaldti* vs *P.luisi* Rodrigues & Moreira, 2022 in the key provided by Rodrigues and Moreira (2022). In *P.luederwaldti*, however, the apical macula of the forewings is elongate-oval and almost reaches the wing apex (Fig. 5A), the male proctiger bears a conical process at base (Rodrigues and Moreira 2016a: fig. 21), and the paramere is not notched at the dorsal surface (Rodrigues and Moreira 2016a: fig. 21). In turn, *P.luisi* is lighter and more yellowish than the other two species (Rodrigues and Moreira 2022: figs 1, 2); has shorter, more rounded, basal forewing maculae that do not surpass the apex of the pronotum (Rodrigues and Moreira 2022: figs 1A, 2A, C); and bears lateral projections on the male proctiger (Rodrigues and Moreira 2022: fig 3C).

**Figure 6.**
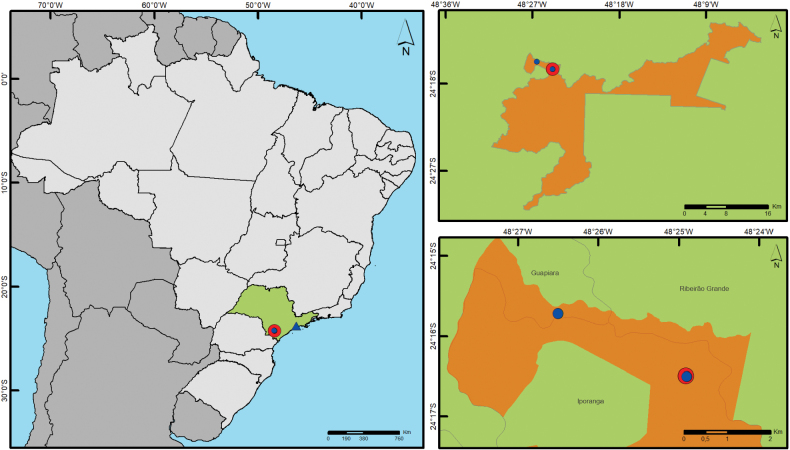
Geographic distribution of *Paraveliaintervalensis* sp. nov. and *P.luederwaldti* Rodrigues & Moreira, 2016 in São Paulo state (highlighted in green). Parque Estadual Intervales is highlighted in orange. *P.intervalensis* sp. nov. record is indicated by a red circle. *P.luederwaldti* records are indicated by blue circles (new) and triangle (previous).

### ﻿New record

#### ﻿ Paravelia luederwaldti

Taxon classificationAnimaliaHemipteraVeliidae

Rodrigues & Moreira, 2016

5CDA3421-CAFE-5567-8919-7F42C11C4CAD

Figs 5 , 6

##### Material examined.

Brazil – **São Paulo** • Iporanga, Parque Estadual Intervales, poças na trilha; 24°16'30"S, 48°24'54"W; 814 m alt.; 23.XI.2023; L.L. Dumas, J.M.S. Rodrigues & R.P.R. Canejo leg.; 2 ♂, 2 ♀, CEIOC 83573 • Guapiara, Parque Estadual Intervales, poças na lama, em estrada de terra; 24°15'43"S, 48°26'30"W; 924 m alt.; 03.XII.2023; L.L. Dumas, J.M.S. Rodrigues, L. Nery & L. Hoehne leg.; 7 ♂, 2 ♀, CEIOC 83574.

This is the first record of this species since its original description by Rodrigues and Moreira (2016a). The type series was also collected in the Serra do Paranapiacaba mountain range, about 220 km north of the new records.

### ﻿Key to the species of Paravelia recorded from Brazil

(modified from Rodrigues and Moreira 2022)

**Table:** 

1	Body shorter than 3.40 mm	**2**
–	Body at least 3.40 mm long	**3**
2	Head, pronotum, legs, and abdomen covered by small black denticles (Rodrigues et al. 2014: fig. 18C, D); anterior lobe of pronotum with a pair of pruinose areas laterally (Rodrigues et al. 2014: figs 18C, D)	***P.splendoris* (Drake & Harris, 1933) [PA, MT, GO, MG, ES**]
–	Body and legs without black denticles, covered by very long setae (Rodrigues et al. 2014: fig. 14C, D); anterior lobe of pronotum without pruinosity (Rodrigues et al. 2014: fig. 14C, D)	***P.capixaba* Moreira, Nessimian & Rúdio, 2010 [AM, PA, MG, ES, RS**]
3	Forewings with bubble-like structures at basal region near humeral angles (Rodrigues et al. 2014: fig. 14A)	***P.bullialata* Polhemus & Polhemus, 1984 [AM, PA, RO**]
–	Forewings without bubble-like structures	**4**
4	Venter of abdomen covered by rounded or oval punctations (Rodrigues and Moreira 2024: fig. 8G)	***P.foveata* Polhemus & Polhemus, 1984 [RR, AM**]
–	Venter of abdomen not covered by rounded or oval punctations	**5**
5	Forewing with an elongated macula basally and a pair of laterally placed rounded maculae apically; all maculae whitish (as in Rodrigues et al. 2014: fig 14B)	**6**
–	Forewing maculae not as above	**7**
6	Body length 3.60–4.20 mm; legs brown, lighter towards base, unarmed; forewing with basal macula long, starting close to humeral angle of pronotum	***P.capillata* (Drake & Harris, 1933) [SE, MT**]
–	Body length 4.55–4.95 mm; legs annulate, with black spinules on femora and tibiae; forewing with basal macula short, starting after apex of pronotum	***P.cognata* (Drake & Harris, 1933) [MA, MT**]
7	Forewing with basal and apical maculae small, of similar size, and rounded (Rodrigues et al. 2014: fig 8A)	***P.micromaculata* Rodrigues,Moreira, Nieser, Chen & Melo, 2014 [PA/MA**]
–	Forewing with basal and apical maculae of different sizes and/or shapes	**8**
8	Pronotal humeral angle with or without spinose projection (Rodrigues and Moreira 2016b: figs 9, 10); forewing with a subtriangular white basal macula along costal margin, at apex a central, oval, whitish area and a pair of irregular maculae on margins (Rodrigues et al. 2014: fig. 19B)	***P.spinifera* Polhemus & Polhemus, 1984 [PA, PA/MA**]
–	Pronotal humeral angle never with spinose projection; forewing maculae not as above	**9**
9	Head, thorax, and abdominal laterotergites covered by small black denticles; pronotum orange or yellowish orange; forewing with an additional white stripe in front of the basal macula; one macula present on cubital vein and a whitish line surrounding the wing; at apex, another white macula (as in Rodrigues et al. 2014: figs 10A, 18B)	**10**
–	Black denticles, if covering body, not distributed as above; pronotum with variable coloration; forewing at most with basal and apical maculae	**12**
10	General body color yellowish orange; body length approximately 5.00 mm; male abdominal sternum VII with a pair of distinct projections (Hungerford 1930: fig. 9)	***P.confusa* (Hungerford, 1930) [AM**]
–	General body color orange to orange-brown; body length 3.80–4.25 mm; male abdominal sternum VII without projections (as in Rodrigues et al. 2014: fig. 10C)	**11**
11	Antennomere IV whitish, with small brown areas on base and apex (Rodrigues et al. 2014: fig. 18B); male proctiger with a distinct horn-like expansion on basal region and small black denticles on apex (Rodrigues et al. 2014: fig 20D); posterior angle of last abdominal laterotergite of female not developed	***P.rotundanotata* (Hungerford, 1930) [PA, MT, MS, MG**]
–	Antennomere IV entirely orange-brown (Rodrigues et al. 2014: fig 10A); male proctiger only with a basal elevation, without small black denticles on apex (Rodrigues et al. 2014: fig. 12B); posterior angle of last abdominal laterotergite of female developed, acute (Rodrigues et al. 2014: fig 12D)	***P.ornata* Rodrigues, Moreira, Nieser, Chen & Melo, 2014 [AM**]
12	Forewing with basal macula distinctly yellow (as in Figs 2A, 4A, 5A)	**13**
–	Forewing with basal macula white or yellowish white	**17**
13	Male abdominal sternum VII with a pair of large projections (Hungerford 1930: fig. 11; Rodrigues and Álvarez Arango 2019: fig. 2D)	**14**
–	Male abdominal sternum VII without projections or lobes	**15**
14	Thorax and abdomen dark brown to black; basal macula of forewing starting from humeral angle; apical macula of forewings, when present, narrow and elongated (Rodrigues et al. 2014: figs 13A, B); hind femur without spines	***P.basalis* (Spinola, 1837) [MG, ES, SP, RJ**]
–	Thorax dark-brown, abdomen ventrally light brown to brown; basal macula of forewing starting close to humeral angle; apical macula of forewings rounded (Rodrigues et al. 2014: fig. 11A); hind femur with spines (Rodrigues et al. 2014: fig 12F; Rodrigues and Álvarez Arango 2019: fig. 3J)	***P.polhemusi* Rodrigues, Moreira, Nieser, Chen & Melo, 2014 [PA, PI, MT**]
15	Apical macula of forewing elongate-oval, almost reaching apex of wing (Figs 5A, 6A); male proctiger with a conical process at base (Rodrigues and Moreira 2016a: fig. 21); paramere without notch on dorsal surface in lateral view (Rodrigues and Moreira 2016a: fig. 21)	***P.luederwaldti* Rodrigues & Moreira, 2016 [SP**]
–	Apical macula of forewing rounded to oval or diamond-shaped, located far from apex of wing (Figs 2A, 4A; Rodrigues and Moreira 2022: figs 1A, 2A, C); male proctiger without conical process at base (Fig. 3C; Rodrigues and Moreira 2022: fig. 3C); paramere with notch on dorsal surface in lateral view (Fig. 3E; Rodrigues and Moreira 2022: fig. 3E)	**16**
16	Legs and venter of abdomen with extensive yellowish areas (Rodrigues and Moreira 2022: figs 1, 2); basal forewing maculae rounded (Rodrigues and Moreira 2022: figs 1A, 2A, C); male proctiger with lateral projections (Rodrigues and Moreira 2022: fig. 3C)	***P.luisi* Rodrigues & Moreira, 2022 [PE**]
–	Legs and venter of abdomen predominantly dark (Figs 2A, 2B, 2D, 4); basal forewing maculae elongated (Fig. 2A, 2D, 4A); male proctiger without lateral projections (Fig. 3C)	***P.intervalensis* sp. nov. [SP**]
17	Body with small black denticles, which can be restricted to male abdominal segment VIII (in *P.cunhai*; Rodrigues and Moreira 2016b: figs 5, 6) or more widespread	**18**
–	Small black denticles completely absent from body	**27**
18	Posterior angle of pronotum ending in a robust, finger-like, upward directed process (Rodrigues and Moreira 2022: fig. 8B); small black denticles on ventral region of head, prosternum, abdominal sterna III–IV, and male abdominal segment VIII; male abdominal sternum VII with a pair of nodules on posterior region (Rodrigues and Moreira 2022: fig. 8C)	***P.digitata* Rodrigues & Moreira, 2016 [RN, PE, BA**]
–	Posterior angle of pronotum without upward directed process; small black denticles with different disposition; male abdominal sternum VII without nodules on posterior region	**19**
19	Posterior angle of pronotum acuminated (as in Rodrigues et al. 2014: fig. 17D); male abdominal sternum VII with a pair of acute projections (as in Moreira and Barbosa 2012: figs 12, 13)	**20**
–	Posterior angle of pronotum rounded (as in Rodrigues et al. 2014: fig. 17B) to slightly acute (as in Rodrigues et al. 2014: fig. 13C), not acuminated; male abdominal sternum VII without projections	**21**
20	Pronotum orange, with anterior lobe, humeral angles, and wide median stripe brownish; base of paramere with dorsal surface slightly widened (Polhemus and Polhemus 1985: fig 2)	***P.truxali* Polhemus & Polhemus, 1985 [GO**]
–	Pronotum dark brown to black, with margins of posterior lobe orange (Rodrigues and Moreira 2022: fig. 10A, C); paramere with a small basal notch on dorsal surface (Rodrigues et al. 2014: fig. 20A)	***P.nieseri* Moreira & Barbosa, 2012 [PI, MG**]
21	Small black denticles present on abdominal laterotergites; body length 3.54–4.00 mm	**22**
–	Small black denticles absent from abdominal laterotergites; body length 4.42–5.52 mm	**24**
22	Body length 3.54 mm; abdominal segment VIII of male without small black denticles; paramere curved mesally, becoming darker and narrower towards apex	***P.nexa* (Drake & Harris, 1933) [MA**]
–	Body length 4.00 mm; abdominal segment VIII of male with small black denticles laterally; paramere not as above	**23**
23	Paramere curved, constricted near base and after middle, with wide apex	***P.kahli* (Drake & Harris, 1933) [MT**]
–	Paramere with apex strongly curved and acute	***P.parilis* (Drake & Harris, 1933) [MT**]
24	Anterior lobe of pronotum laterally with a pair of silvery pubescent areas (Rodrigues et al. 2014: fig. 2A, B); abdominal sterna covered by small black denticles	***P.bahiana* Rodrigues, Moreira, Nieser, Chen & Melo, 2014 [BA**]
–	Anterior lobe of pronotum without silvery pubescence (as in Rodrigues et al. 2014: figs 7A, 17B); abdominal sterna with or without small black denticles	**25**
25	Body length approximately 5.50 mm; forewing with a white, tear-like, basal macula starting from below humeral angle and surpassing posterior margin of pronotum (Rodrigues et al. 2014: fig. 7A)	***P.lacrymosa* Rodrigues, Moreira, Nieser, Chen & Melo, 2014**
–	Body length 4.42–5.15 mm; forewing with a white, oval, basal macula not reaching humeral angle and ending adjacent to posterior margin of pronotum or slightly beyond (Rodrigues et al. 2014: fig. 17B; Rodrigues and Moreira 2016b: fig. 1)	**26**
26	Grasping comb occupying 1/5 of fore tibial length; hind femur with a row of spines (Rodrigues and Moreira 2016b: fig. 4); abdominal sterna II–VI without black denticles (Rodrigues and Moreira 2016b: fig. 2)	***P.cunhai* Rodrigues & Moreira, 2016 [PA**]
–	Grasping comb occupying 1/3 of fore tibial length; hind femur without spines; abdominal sterna II–VI with black denticles	***P.lanemeloi* Moreira & Barbosa, 2012 [MG**]
27	Body at least 4.90 mm long; non-bromelicolous species	**28**
–	Body shorter than 4.90 mm; bromelicolous species	**31**
28	Posterior angle of pronotum ending in a digitiform process (Rodrigues et al. 2014: fig. 3F; Rodrigues and Moreira 2016b: fig. 8); male abdominal sternumVII without projections or lobes on posterior margin	**29**
–	Posterior angle of pronotum rounded (as in Rodrigues et al. 2014: fig. 19A, C); male abdominal sternum VII with a pair of projections (Rodrigues et al. 2014: fig. 19D) or with two small lobes medially on posterior margin (Hungerford 1930: fig. 4)	**30**
29	Body at most 6.00 mm long; forewing with apical macula projected laterally in the distal region (Rodrigues and Moreira 2016b: fig 8); paramere slightly narrowed at base, without dorsal notch (Polhemus and Polhemus 1984: fig 5)	***P.juruana* Polhemus & Polhemus, 1984 [AM**]
–	Body longer than 6.00 mm; forewing with apical macula evenly ovate, not projected laterally (Rodrigues et al. 2014: fig 13C); paramere slightly curved, with a dorsal notch near base (Spangler 1989: figs 6, 8)	***P.biae* Spangler, 1989 [PA, RO**]
30	Body length 5.80–6.00 mm; femora yellowish at basal half, rest of legs brownish (Rodrigues et al. 2014: fig 19C); male with a pair of distinct projections on posterior margin of abdominal sternum VII (Rodrigues et al. 2014: fig 19D)	***P williamsi* (Hungerford, 1930) [AM**]
–	Body length approximately 5.00 mm; legs dark brown (Rodrigues et al. 2014: fig 19A); posterior margin of male abdominal sternum VII medially extended, forming two small lobes on sides of a central concavity (Hungerford 1930: fig 4)	***P.platensis* (Berg, 1883) [SP**]
31	Anterior lobe of pronotum without pubescence laterally (Rodrigues and Moreira 2016a: fig. 29)	***P.itatiayana* (Drake, 1951) [SP, RJ**]
–	Anterior lobe of pronotum with a pair of lateral, white, pubescent areas (Rodrigues and Moreira 2016a: figs 1, 3–5, 27, 30) (some specimens of *P.recens* have the pubescence irregular and not so evident; Rodrigues et al. 2014: fig 18A)	**32**
32	Pubescence on anterior lobe of pronotum narrow, elongate, slightly curved laterally (Rodrigues and Moreira 2016a: fig. 27); distance between basal and apical maculae of forewing approximately 1/3 the length of apical macula (Rodrigues and Moreira 2016a: fig. 27)	***P.gabrielae* Moreira & Barbosa, 2011 [ES, SP**]
–	Pubescence on anterior lobe of pronotum subtriangular (Rodrigues and Moreira 2016a: figs 1, 3–5), rectangular (Rodrigues and Moreira 2016a: fig 30), or irregular (Rodrigues and Moreira 2016a: fig 48); distance between basal and apical maculae of forewing equal to or longer than the length of apical macula (Rodrigues and Moreira 2016a: figs 1, 3–5, 30, 48)	**33**
33	Body black (Rodrigues and Moreira 2016a: fig 30); pubescence on anterior lobe of pronotum rectangular (Rodrigues and Moreira 2016a: fig 30); male proctiger with two or three elevations on dorsal surface (Rodrigues and Moreira 2016a: fig 32); paramere as in Rodrigues and Moreira (2016a: fig 32)	***P.manausana* Polhemus & Polhemus, 1984 [AM**]
–	Body brown, reddish brown, or orange-brown (Rodrigues and Moreira 2016a: figs 1, 3–5, 48); pubescence on anterior lobe of pronotum subtriangular (Rodrigues and Moreira 2016a: figs 1, 3–5) or irregular (Rodrigues and Moreira 2016a: fig 48); male proctiger with at most one dorsal elevation plus a small acute process (Rodrigues and Moreira 2016a: figs 12, 15, 41); paramere not as above	**34**
34	Apical macula of forewing usually rounded or oval, shorter than basal macula (Rodrigues and Moreira 2016a: fig 48); male proctiger without elevation or acute process anteriorly (Rodrigues and Moreira 2016a: fig 41)	***P.recens* (Drake & Harris, 1935) [AM, PA, ES**]
–	Apical macula of forewing narrow, elongate, at least as long as basal macula (Rodrigues and Moreira 2016a: figs 1, 3–5); base of male proctiger with a weak elevation (Rodrigues and Moreira 2016a: fig 12) or a weak elevation plus small acute process (Rodrigues and Moreira 2016a: fig 15)	**35**
35	Male proctiger only with a weak elevation at base (Rodrigues and Moreira 2016a: fig 12); paramere as in Rodrigues and Moreira (2016a: fig 12)	***P.bachmanni* Rodrigues & Moreira, 2016 [SP, SC**]
–	Male proctiger with a weak elevation plus a small acute process at base (Rodrigues and Moreira 2016a: fig. 15); paramere as in Rodrigues and Moreira (2016a: fig 15)	***P.bromelicola* Rodrigues & Moreira, 2016 [SP, SC**]

## Supplementary Material

XML Treatment for Paravelia intervalensis

XML Treatment for Paravelia luederwaldti

## ﻿Additional information

### Author ORCIDs

Evaldo Alves Joaquim https://orcid.org/0009-0001-6660-8727

Juliana Mourão dos Santos Rodrigues https://orcid.org/0000-0003-2872-138X

Felipe Ferraz Figueiredo Moreira https://orcid.org/0000-0002-6692-0323

Leandro Lourenço Dumas https://orcid.org/0000-0002-6590-3388

### Data availability

All of the data that support the findings of this study are available in the main text.

## References

[B1] Furlan SA Leite SA (2008) Plano de manejo do Parque Estadual de Intervales. https://fflorestal.sp.gov.br/planos-de-manejo/planos-de-manejo-planos-concluidos/plano-de-manejo-pe-intervales/ [Accessed on 19.X.2024]

[B2] Hungerford HB (1930) Three new *Velia* from South America. Journal of the Kansas Entomological Society 3: 23–27. 10.1093/aesa/23.1.120

[B3] IBGE (2024) Cidades e Estados. https://www.ibge.gov.br/cidades-e-estados [Accessed on 19.X.2024]

[B4] Mazzucconi S López-Ruf M Bachmann AO (2009) Hemiptera – Heteroptera: Gerromorpha y Nepomorpha. In: Dominguez E Fernández HR (Eds) Macroinvertebrados bentónicos sudamericanos.Sistemática y Biología. Fundación Miguel Lillo, Tucumán, 167–231.

[B5] Moreira FFF (2024) Veliidae in Catálogo Taxonômico da Fauna do Brasil. http://fauna.jbrj.gov.br/fauna/faunadobrasil/9453 [Accessed on 09.XI.2024]

[B6] Moreira FFF Barbosa JF (2012) Two new species of *Paravelia* Breddin, 1898 and distributional notes concerning the Veliidae from Minas Gerais State, Brazil (Insecta: Hemiptera: Heteroptera: Gerromorpha). Zootaxa 3354: 58–68. 10.11646/zootaxa.3354.1.2

[B7] Polhemus DA (2021) *Callivelia*, a new genus for certain Neotropical Veliinae (Heteroptera: Veliidae), including description of a new species. Zootaxa 4950(2): 345–360. 10.11646/zootaxa.4950.2.6 33903441

[B8] Polhemus JT Polhemus DA (1984) Notes on Neotropical Veliidae (Hemiptera) IX: additional new species of *Paravelia* from South America. Amazoniana 8: 497–504.

[B9] Polhemus JT Polhemus DA (1985) Studies on Neotropical Veliidae (Hemiptera) VIII: new species and notes. Pan-Pacific Entomologist 61: 163–169.

[B10] Polhemus JT Polhemus DA (1991) A review of the Veliid fauna of bromeliads, with a key and description of a new species (Heteroptera: Veliidae). Journal of the New York Entomological Society 99: 204–216.

[B11] Polhemus JT Polhemus DA (1993) Two new genera for New World Veliinae (Heteroptera: Veliidae). Journal of the New York Entomological Society 101: 391–398.

[B12] Polhemus DA Molano F Morales I Moreira FFF Floriano CFB (2019) *Altavelia*, a new genus of Neotropical Veliinae (Heteroptera: Gerromorpha: Veliidae), with a key to species and descriptions of four new species from Colombia. Zootaxa 4585(2): 295–314. 10.11646/zootaxa.4585.2.4 31716169

[B13] Ribeiro MC Metzger JP Martensen AC Ponzoni FJ Hirota MM (2009) The Brazilian Atlantic Forest: how much is left, and how is the remaining forest distributed? Implications for conservation. Biological Conservation 142: 1141–1153. 10.1016/j.biocon.2009.02.021

[B14] Rodrigues HDD Álvarez Arango LF (2019) A new species of *Oiovelia* from Colombia, with notes for other three species of South American Veliinae (Heteroptera: Gerromorpha: Veliidae). Papéis Avulsos de Zoologia 59: e20195935. 10.11606/1807-0205/2019.59.35

[B15] Rodrigues HDD Moreira FFF (2016a) Four new species of *Paravelia* (Hemiptera: Heteroptera: Veliidae) from Brazil, with notes on previously described species. Canadian Entomologist 148: 642–667. 10.4039/tce.2016.18

[B16] Rodrigues HDD Moreira FFF (2016b) A new species, new synonymy, and notes on *Paravelia* Breddin (Hemiptera: Heteroptera: Veliidae). Papéis Avulsos Zoologia 56: 183–188. 10.11606/0031-1049.2016.56.17

[B17] Rodrigues JMS Moreira FFF (2022) New species, new records, and updated key to the *Paravelia* (Hemiptera: Heteroptera: Veliidae) from Brazil. Insects 13: 541–558. 10.3390/insects13060541 35735878 PMC9224760

[B18] Rodrigues HDD Moreira FFF (2024) *Foveavelia*, a new South American genus of Veliinae (Hemiptera: Heteroptera: Veliidae). PeerJ 12: e16772. 10.7717/peerj.16772 PMC10961057 38525284

[B19] Rodrigues HDD Moreira FFF Nieser N Chen PP Melo AL Dias-Silva K Giehl NFS (2014) The genus *Paravelia* Breddin, 1898 (Hemiptera: Heteroptera: Veliidae) in Brazil, with descriptions of eight new species. Zootaxa 3784: 1–47. 10.11646/zootaxa.3784.1.1 24872030

[B20] Spangler PJ (1989) A new species of Neotropical water bug, *Paraveliabiae*, from Brazil (Heteroptera: Veliidae). Proceedings of the Entomological Society of Washington 91: 360–366.

